# Factors shaping the decision-making process to continue or discontinue antipsychotics: exploratory qualitative study of 12 individuals in remission from first-episode psychosis

**DOI:** 10.1192/bjo.2025.10817

**Published:** 2025-09-04

**Authors:** Laurent Béchard, Elizabeth Anderson, Olivier Corbeil, Maxime Huot-Lavoie, Sébastien Brodeur, Amal Abdel-Baki, Charles Massé, Mina Gabriel-Courval, Marie-Ève Coté, Annie LeBlanc, Marc-André Roy, Marie-France Demers, Sophie Lauzier

**Affiliations:** Faculty of Nursing, Université Laval, Quebec, Canada; Faculty of Pharmacy, Université Laval, Quebec, Canada; School of Psychology, Faculty of Social Sciences, Université Laval, Quebec, Canada; Faculty of Medicine, Université Laval, Quebec, Canada; Department of Psychiatry and Addictions, University of Montréal, Montreal, Canada; Quebec Mental Health University Institute, CIUSSS-CN, Quebec, Canada; Clinical Pharmacist, Quebec Mental Health University Institute, CIUSSS-CN, Quebec, Canada; CAP – Rétablissement, CERVO Brain Research Centre, Quebec, Canada; VITAM - centre de recherche en santé durable, CIUSSS-CN, Quebec, Canada; CHU de Québec-Université Laval Research Centre, Quebec, Canada

**Keywords:** Qualitative research, psychotic disorders/schizophrenia, decision-making, antipsychotics, recovery

## Abstract

**Background:**

The decision-making process regarding antipsychotic continuation or discontinuation following remission from first-episode psychosis (FEP) remains complex and underresearched. While discontinuation increases the risk of relapse, concerns over long-term side-effects such as metabolic disturbances and extrapyramidal symptoms also exist. Current guidelines recommend maintaining antipsychotics for 1–5 years, emphasising shared decision-making (SDM) between clinicians and patients.

**Aims:**

This study aimed to explore the decision-making process and describe the factors influencing the decision to discontinue or continue antipsychotic treatment following remission from FEP, from the patients’ perspective.

**Method:**

A descriptive qualitative study was conducted with 12 individuals in remission from FEP who received care at early intervention services in Quebec, Canada. Data were collected through online semi-structured interviews and analysed thematically to identify key factors influencing treatment decisions.

**Results:**

The decision-making process was activated by treatment reflection triggers and shaped by various perceptions (of illness, treatment and stigma) and relationships (with friends, family and the clinical team), ultimately leading to decisions to either discontinue, continue (at standard or reduced dose) or remain ambivalent. This dynamic process was guided by participants’ motivators, such as well-being and societal contribution. Most participants felt that discontinuation discussions were not initiated by the clinical team.

**Conclusions:**

The decision-making process is driven by motivators that were found to be linked to the concept of personal recovery. This study highlights the need for proactive, personalised discussions between clinicians and patients. Future research should focus on decision aids tailored to the FEP population to support SDM and improve treatment outcomes.

The duration of antipsychotic treatment following remission from a first episode of psychosis (FEP) remains the subject of debate.^1,2^ While discontinuation can increase the risk of psychotic relapse,^3–5^ concerns also exist about potential side-effects such as metabolic disturbances^6,7^ and extrapyramidal symptoms associated with continuation.^8^ Observational studies suggest that long-term antipsychotic use may reduce mortality,^9^ but its overall impact on long-term recovery remains unclear.^10^ Current evidence on long-term outcomes, such as functional recovery following antipsychotic discontinuation, remains limited and conflicting, with only two open-label extension studies from randomised controlled trials (RCTs) (one spanning 7 years and the other 10 years, and providing divergent results).^11,12^ Current guidelines worldwide recommend waiting from 1 to 5 years post-remission before considering antipsychotic discontinuation.^3–5^ These guidelines also emphasise the importance of shared decision-making (SDM), a process by which clinicians and patients collaborate to make decisions based on both clinical evidence and patient preferences.^4,5,13^ Additionally, existing guidelines provide few specifics on how to effectively implement SDM in navigating the complexities of decisions such as antipsychotic discontinuation, and its application remains limited in practice.^14–17^

Previous qualitative studies have examined patient perspectives on shared decision-making and treatment with antipsychotics following FEP;^18,19^ however, research specifically focusing on the decision-making process for antipsychotic discontinuation following long-term remission remains limited.^20^ A better understanding of this process could help clinicians guide discussions more effectively, allowing patients to share their values and preferences, ultimately improving SDM and aligning treatment decisions with what matters most to the patient.

This article aims to explore the decision-making process and describe the factors influencing the decision to discontinue or continue antipsychotic treatment following remission from FEP, from the patients’ perspective. By shedding light on the decision-making process, we aim to give patients a voice in expressing what they consider important in treatment decisions, thereby offering clinicians valuable insights to support patient-centred care.

## Method

### Study design

A descriptive qualitative study using semi-structured interviews was conducted to gain in-depth knowledge on the decision-making process and factors influencing the choice to discontinue or continue antipsychotic treatment following FEP remission, from the perspective of people under care, in two early intervention services (EIS) in Quebec, Canada.^21^ The authors assert that all procedures contributing to this work comply with the ethical standards of the relevant national and institutional committees on human experimentation, and with the Helsinki Declaration of 1975 as revised in 2013. All procedures involving human subjects/patients were approved by CÉR-S en neurosciences et santé mentale du CIUSSS-CN (no. MP-13-2021-2325). Study procedures were explained to participants, and informed written consent was obtained. The reporting of this study adheres to the consolidated criteria for reporting qualitative research (COREQ).^22^

### Setting and participants

This study was conducted in the two largest EIS in the province of Quebec (Canada), where a total of 200 individuals with FEP are admitted each year and followed for 3 years on a case management basis. These EIS serve young adults aged 18 to 35 years with a primary diagnosis of non-affective or affective psychosis and who have never been treated, or have been treated intermittently for 6 months at most. Recruitment for this study took place from July 2021 to June 2023. Inclusion criteria were: (a) a primary diagnosis of a schizophrenia spectrum or other psychotic disorder; bipolar disorder with psychotic features; major depression with psychotic features (DSM-5); (b) at least 18 months of follow-up in EIS (the minimum recommended duration of antipsychotic treatment according to Canadian guidelines^5^); (c) stable condition at recruitment (no acute psychotic symptoms) as assessed by the case manager or psychiatrist; and (d) proficiency in the French language. Exclusion criteria were: (a) under an involuntary treatment order; (b) potential harm from discussing treatment changes as assessed by the case manager or psychiatrist; and (c) use of clozapine, because the issue of treatment discontinuation might be very different in such instances. Based on inclusion/exclusion criteria, potential participants were identified by case managers who briefly explained the study and obtained agreement to be contacted by a member of the research team. Based on healthcare providers’ description, participants were purposefully selected to capture a diverse, information-rich sample, considering sociodemographic characteristics (gender, ethnicity) and treatment decisions (continuation, discontinuation) to reflect the overall decision-making process.^23^ Potential participants were contacted via telephone by a male clinical pharmacist-PhD student (L.B., the lead author), who was not part of the clinical team. L.B. explained the study and obtained consent. Participants received a Can$40 gift card for their time.

### Data collection

Data were collected using semi-structured interviews. The interview guide was developed by an interdisciplinary research team, including a researcher trained in qualitative research in health sciences (S.L.), a SDM expert (A.L.), two clinical pharmacists specialised in mental health (M.-F.D. and L.B.), a psychiatrist (M.-A.R.) and a patient partner in research (M.-E.C.). The interview guide was developed according to the Ottawa Decision Support Framework (ODSF)^24^ and the Connectedness, Hope, Identity, Meaningfulness, Empowerment (CHIME) recovery framework,^25^ and was informed by a broad literature review on SDM and antipsychotic medication.^18,19,26–29^ Briefly, the interview guide covered key topics including the participant’s understanding of medication, perceptions of mental illness, communication with the clinical team, SDM regarding the decision to discontinue or maintain antipsychotics, barriers to decision-making (ODSF) and the application of the CHIME framework to the decision-making process of discontinuation or continuation. The interview guide also consisted of questions on basic demographic information including age, gender, marital status, educational level, occupation and living arrangements. It was pilot tested with clinicians (*n* = 3) and a patient partner in research. Minor adjustments were made following the pilot test. The interview guide was also piloted with the first three participants and, since only minor changes were needed, the data collected were included in the analysis. The final interview guide is available in the Supplementary materials, available at https://doi.org/10.1192/bjo.2025.10817.

All interviews were conducted online via Microsoft Teams (for Windows; Microsoft Corp., Redmond, WA, USA; www.microsoft.com/teams) by L.B., who received training on qualitative interviewing from a senior psychiatrist (M.-A.R.) and an expert in qualitative research (S.L.). Only L.B. and participants were present during the interviews, which were conducted in French. Video recordings were made, converted to audio and transcribed verbatim by health science undergraduate students (E.A., C.M. and M.G.-C.). Transcripts were reviewed for accuracy by L.B. No repeat interviews were conducted, and field notes were taken after each interview. Except for one participant who had previously collaborated with the lead author as a patient partner in research on a separate project, there was no prior relationship between the interviewer and participants. Transcripts were not returned to participants for comment or correction.

### Data analysis

The transcripts were thematically analysed using a codebook developed through a continuous validation process.^30^ Three team members (S.L., L.B. and E.A.) independently coded each interview line by line, following a process inspired by thematic analysis, where codes were derived from ODSF, CHIME, the literature, the interview guide and the data.^31^ Memos were used throughout this process to capture reflections and ideas. The initial coding informed the creation of a preliminary codebook, which was reviewed and cross-checked by a qualitative research expert (S.L.) and a clinical pharmacist specialised in mental health (M.-F.D.).

This initial codebook was then tested on two additional interviews by L.B. and E.A., leading to the development of a revised version. Using this version of the codebook and allowing for the emergence of new codes from the data, a total of 50% of transcripts were randomly double-coded by L.B. and E.A. using NVivo Pro (version 12 for Windows; Lumivero, Denver, CO, USA; https://lumivero.com/products/nvivo/) to ensure intercoder reliability. Following coding, codes were organised into categories and then grouped into broader themes through a consensus-building process among the three researchers (L.B., S.L. and E.A.). Emerging themes and relationships were identified based on code summaries. Feedback on the findings was provided by one participant, and the credibility and transferability of the results were assessed through discussions with a patient partner and all members of the research team. Participants’ quotations presented in this manuscript were translated into English by a certified translator. Pseudonyms were used for participants. To capture a variety of perceptions, we initially planned on recruiting at least 20 participants, but interviews were conducted until data saturation was reached.^32^

## Results

**Fig. 1:**
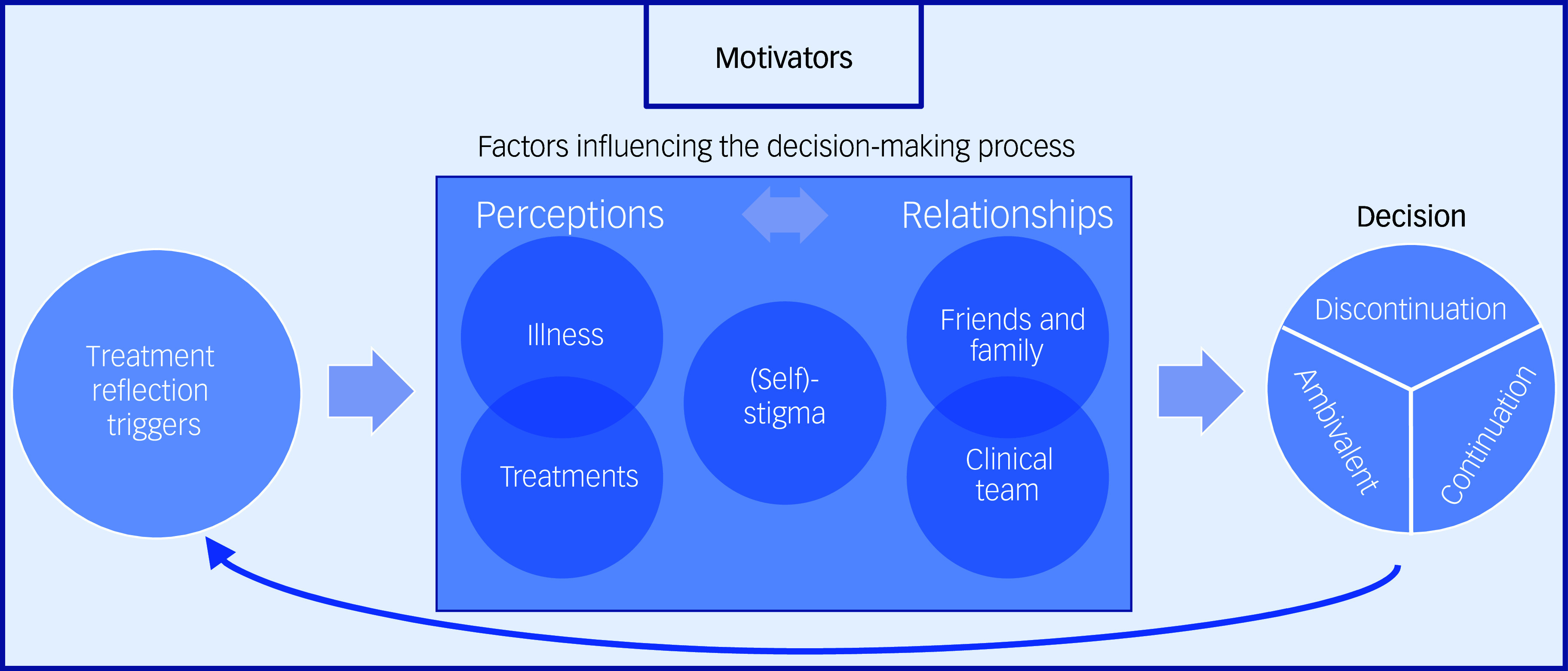
The decision-making process.

Of the 40 individuals identified by the clinical team, 23 agreed to be contacted for potential participation and, ultimately, 12 completed the qualitative interview, with no dropouts. Nine individuals who had initially agreed to be contacted were potentially eligible, but could be reached, did not proceed: three could not have their eligibility confirmed following significant project delays; four had not discussed antipsychotic discontinuation with their clinical team (the key focus of the study); and two were not interviewed due to their profile and the need to prioritise diversity. Most were single White men averaging 26 years of age (see Table 1). Interviews averaged 58 min (range, 39–95 min). The decision-making process was activated by treatment reflection triggers and shaped by various perceptions (of illness, treatments and stigma) and relationships (with friends and family and the clinical team), ultimately leading to a decision of whether to discontinue, continue or remain ambivalent about this decision. The entire decision-making process is guided by participants’ personal motivators (see Fig. 1).

**Table 1:** Demographic characteristics of participants (*n* = 12)

Characteristic	Value
Mean age, years (s.d.)	26.25 (5.1)
Gender, *n* (%)	
Male	7 (58.3)
Ethnicity,^a^ *n* (%)	
Black	1 (8.3)
White	11 (91.7)
Educational level, *n* (%)	
High school	5 (41.7)
Vocational diploma	1 (8.3)
CEGEP^b^	3 (25)
University	3 (25)
Employment status, *n* (%)	
Employed	5 (41.7)
Stay-at-home parent	1 (8.3)
Student	4 (33.3)
Unemployed	2 (16.7)
Marital status, *n* (%)	
Common-law partner	1 (8.3)
Single	11 (91.7)
Living situation (%)	
House	1 (8.3)
Independent apartment	5 (41.7)
Parental home	3 (25)
Room with or without board	2 (16.7)
Student housing	1 (8.3)
Median follow-up, months (range)	30.4 (22.8–192.2)
Median number of hospitalisations, *n* (range)	1 (0–5)
Insight into illness,^c^ *n* (%)	6 (50)
Decision regarding medication, *n* (%)	
Ambivalent	2 (16.7)
Continuation	7 (58.3)
Discontinuation	2 (16.7)
Discontinuing	1 (8.3)

a Self-identified ethnicity.

b CEGEP is a Quebec college providing pre-university and vocational education.

c Clinical insight was assessed through self-reported mental health illness.

### Decision: continue, discontinue or ambivalent

At the time of the interviews, participants’ decisions on antipsychotic use varied: seven had opted to continue, including one who reduced the dose; two were uncertain about whether to continue or stop; one was in the process of discontinuing; and two had already stopped. One participant described how he perceived the benefits of continuing: ‘Because for now, the medications are working, and there’s clearly a positive effect when you weigh the pros and cons [of continuing medication]’ (Noah, continued medication). In contrast, another participant who stopped medication emphasised the desire for independence: ‘[…] for me, it was better to stop and see how I can function on my own […] rather than just continuing to be sedated my whole life […]’ (Olivia, stopped medication). Finally, another participant was ambivalent: ‘I’m not ready to make the decision right now, anyway, so that’s basically it: I’m going to keep gathering a bit more information […]’ (Isabella, ambivalent). It was acknowledged that the decision-making process is not static and could restart following new treatment reflection triggers, prompting individuals to reconsider whether to continue or stop antipsychotic treatment: ‘Um, in 10 years, maybe [I’ll stop], because by then I might have stopped using [drugs], and I’ll be at a different point in my life’ (Henry, continued medication).

### Treatment reflection triggers

Except for the one case where the clinician initiated the discussion, the triggers for participants’ reflections on treatment were diverse. For some, this reflection began during significant life transitions, such as finishing studies, which symbolised a desire to close one chapter and move on to the next: ‘The fact that I’m finishing my studies soon has also pushed me a bit to try to reduce [my medication] to, as you said, close this chapter and move on to something else afterward’ (James, discontinuing medication). For others, the reflection was more closely tied to their illness or treatment trajectory, such as the conclusion of EIS, which could limit access to specialised mental healthcare: ‘[…] I think it will be better for me to have access to a psychiatrist because eventually, if I want to stop [my medication], my family doctor isn’t comfortable with [discontinuing medication], so that’s when we talked about it’ (Isabella, ambivalent). Other triggers included prolonged illness stability, concerns about long-term side effects and adverse effects affecting quality of life or life goals such as sedative adverse effects that hindered pursuing life goals: ‘[…] with my medication, I really have a hard time getting up early, and I really want to teach. I really care about my job. I feel good at the school and everything. […] So, the idea of reducing my dose came into play … with the goal of reducing it, so that I could get to school’ (Emma, reduced medication). Discussion with friends or family could also trigger the process: ‘[My mom] suggested the idea [of discontinuing my medication]. I thought about it, I mean, I gave it some thought afterward. I thought it was a good idea. I talked to my psychiatrist about it, and it all just happened after that’ (James, discontinuing medication).

Many participants (*n* = 6) reported that discussions about the choice to continue or discontinue antipsychotics never occurred with their clinical team. Two of these participants were planning to initiate this conversation in the near future. Among those who had discussed discontinuation (*n* = 6), all but one had initiated the conversation themselves, suggesting that it might not have occurred otherwise: ‘Yeah, I was the one [who brought up the topic of stopping antipsychotics]. It was actually me, because no one else would have brought it up if it wasn’t for me’ (Liam, stopped medication).

### Motivators in the decision-making process

Participants identified motivators as central to their decision-making process, citing desires such as achieving well-being, contributing to society and living a normal life. These motivators acted as the underlying drivers for engaging in treatment-related decisions. Notably, while some participants shared similar motivators, these did not necessarily lead to the same decisions regarding stopping or continuing the treatment.

Well-being was the primary motivator for most participants (*n* = 8). Interviewer: ‘What is most important to you when making this decision?’ Interviewee: ‘Well, my well-being, I would say, that’s definitely it’ (Liam, stopped medication). However, participants had different conceptions of well-being. For one participant who discontinued treatment, well-being was expressed as a broader sense of happiness. In contrast, another participant who chose to continue treatment also centred his decision around well-being but defined it more narrowly in terms of illness management, particularly the absence of symptoms: ‘I don’t have weird thoughts […] I don’t think I’m God, I’m not paranoid. I’m just normal, and I’m really happy to be … healthy, so I tell myself, “If it’s not hurting me, why stop?’” (Daniel, continued medication).

Another key motivator was societal contribution (*n* = 3), which was expressed as encompassing maintaining employment, contributing to society and engaging in a social life. One participant explained: ‘[My psychiatrist’s] fear was that I would relapse [if we reduced my medication], and I also had that fear to some extent. However, my desire to go back to work was stronger than my fear’ (Emma, reduced medication). This desire influenced her decision to continue treatment, but at a minimal dose, balancing the risk of relapse with her goal of returning to work.

The last motivator identified for engaging in the decisional process was to regain a sense of normalcy. Interviewer: ‘What is most important to you when making this decision?’ Interviewee: ‘[…] I’m thinking that [stopping my medication] would help me get back to a normal life’ (James, discontinuing medication).

### Factors influencing the decision-making process

Multiple factors were mentioned as influencing the decision-making process; these include perceptions of the illness and treatment, as well as relationships with friends and family and the clinical team. Stigma, both self-stigma and social stigma, was another contributing factor that resulted from patients’ perceptions and their relationships.

#### Illness perceptions

Participants indicated that perceptions of the risk of relapse and its potential consequences played a key role in their decision-making process. Among participants, half viewed the risk of relapse as very high. The perceived consequences of relapse varied: some saw it as minor (*n* = 5), expecting quick symptom recognition and resolution with restarting treatment; others saw it as severe (*n* = 4), fearing schizophrenia, job loss or social problems, while some were unsure (*n* = 3). Ome participant chose to continue treatment, driven by a desire to avoid relapse and its potential consequences linked to antipsychotic discontinuation: ‘I almost lost my job. I said a lot of hurtful things to my friends and family, and my husband’s perception of me was deeply affected. […] I’ve been lucky in that regard, but I think a lot of people, when they experience psychosis, they lose many people around them, including their partner’ (Sophia, continued medication).

The perceived causes of psychosis, which can influence the decision-making process, vary and are multiple. Some participants who attributed their psychosis to external factors such as substance use (e.g. alcohol, drugs) or stress (e.g. COVID-19 isolation, police brutality) viewed their risk of relapse as minimal and expressed the opinion that they were more inclined to discontinue treatment: ‘[…] in my case, I think that if I manage to quit cannabis, there would be no reason that I would relapse’ (James, discontinuing medication). In contrast, those who attributed their psychosis to internal factors, such as genetics, were more cautious and likely to continue treatment.

#### Treatment perceptions

Treatment perceptions played a significant role in the decision-making process. Participants who saw antipsychotics as essential for controlling psychotic symptoms were more likely to continue their treatment. In contrast, those who believed that alternative approaches, such as meditation, could maintain stability were more inclined to consider discontinuation (*n* = 3): ‘If I’ve reached [a stable condition], if we reduce the medication, we can try to maintain that stability through the environment, instead of relying on the medication’ (Liam, stopped medication). While pharmacological treatments were recognised for reducing relapse risk and managing symptoms, nearly all participants (*n* = 10) had experienced or were aware of significant adverse effects, such as fatigue (*n* = 3), weight gain (*n* = 3), tremors (*n* = 1), sexual dysfunction (*n* = 2) and emotional blunting (*n* = 1). These adverse effects often pushed participants to consider treatment discontinuation: ‘[…] ever since my medication, I’ve gained a lot of weight, and I can’t lose it. […] if I’m not careful, I won’t be able to keep it off […]’ (Isabella, ambivalent).

#### Stigma and self-stigma

Both social and self-stigma influenced participants’ decisions about whether to continue or discontinue antipsychotic treatment. Diagnostic-related stigma appeared when participants internalised negative stereotypes about mental illness. One participant questioned his schizophrenia diagnosis, not seeing himself fit his own stigmatising view of the illness: ‘Yeah, it was the name that freaked me out. Schizophrenic, just hearing that […] you know, I’m a pretty chill guy, I can interact with people, you know, I’m not going around digging through the trash like I see some people do sometimes. […] I don’t want to turn into a good-for-nothing like [people with schizophrenia]’ (Henry, continued medication). These stigmatising beliefs about schizophrenia affected his sense of identity and contributed to his doubts about the diagnosis. Though still sceptical he decided to continue medication, driven by the need to avoid a substance-induced psychosis, which he recognised as a real risk due to his drug use. For others, medication itself was a reminder of illness, contributing to low self-esteem and a sense of living an abnormal life (*n* = 4; all took their medication orally), which could have influenced their decision to discontinue treatment: ‘For me personally, taking medication is really a burden; […] it means that I’m sick. […] I have an illness, and that really affects my self-esteem […] because it’s like I’m putting myself down, without meaning to’ (Lily, ambivalent). ‘[When you take medication] you know, it feels like you always have some kind of band-aid on, it’s like … you’re still healing from something, you know?’ (Olivia, stopped medication). Additionally, two participants expressed discomfort with being medicated or feeling dependent on medication, which could also have influenced their decision to consider discontinuation.

#### Relationships with friends and family

In all cases, friends and family were not directly involved in the decision-making process because they were not physically present during discussions with the clinical team when decisions were made: ‘I’m the one who will make the decision, not [my friends or family], definitely not. […]. I’m the one steering the ship here. It’s my mind, not theirs’ (Ethan, continued medication). However, the friends and family could still have had a significant influence (*n* = 10), because participants often sought advice or support or were indirectly influenced by the opinions of those close to them. For example, the fear of psychotic relapse from parents can shape how participants viewed psychosis and their decisions. One participant that continued stated: ‘Well, my mom didn’t want me to stop because she didn’t want me to relapse. […] My mother was more afraid than anything else, she was more worried. […] I guess now, I’m a bit scared of relapsing, too’ (Daniel, continued medication). In some cases, conflicting opinions with friends and family (*n* = 3) created tension regarding the decision to stop or continue antipsychotics: ‘Well, of course, I’d like [my brother] to … not realise, but maybe change his point of view a little. Um, it definitely affects me in the sense that I’d like him to stop telling me to stop taking my medication, because it causes a bit of unnecessary friction’ (Sophia, continued medication). In contrast, others felt confident that their loved ones would support them regardless of their decision (*n* = 3): ‘[…] people around me, I think they’ll support me in this, whether I stop or continue’ (Isabella, ambivalent).

#### Relationships with the clinical team

The clinical team played a significant role in decision-making, with many participants expressing trust and satisfaction in their relationship, often viewing the team as compassionate and supportive. The psychiatrist’s opinion was especially influential for some (*n* = 4), making participants more inclined to follow their expert advice regarding antipsychotic use: ‘[My psychiatrist] knows the substance best […] He’s been working at the hospital for years, treating patients, so I think he’s probably the best person to trust when it comes to medication’ (Olivia, stopped medication). Trust in the psychiatrist often led these participants to continue their treatment. However, others faced challenges in communication, feeling unable to speak freely or share their opinions (*n* = 3). Poor communication often delayed the decision-making process, with antipsychotics typically being continued until participants felt sufficiently comfortable to raise the issue of discontinuation. However, starting the decision-making process did not always lead to discontinuation, because outcomes varied: one participant continued, another discontinued and one remained ambivalent, as expressed by this participant: ‘Now, I feel intimidated in an environment full of people who have PhDs, Master’s degrees and all that, in things that I don’t know anything about. But they don’t have a PhD in my body’ (Isabella, ambivalent).

## Discussion

Our study highlights the complexity of the decision-making process surrounding antipsychotic continuation or discontinuation following remitted FEP. Multiple factors influence this process, not only varying between individuals but also in their perceived importance, which shifts over time. The decision-making process is dynamic, with individuals revisiting their choices as new triggers arise. At the core of this process are the personal motivators that guide decisions. The motivators identified in this study – living a normal life, well-being and contributing to society – are directly related to the concept of personal recovery, which involves a shift in attitudes and perceptions towards achieving a fulfilling life despite the challenges posed by mental illness.^25^ These motivators align seamlessly with the CHIME framework, which outlines the key components of personal recovery.^25^ For instance, the desire for well-being and living a normal life corresponds to hope and meaning in the CHIME model, while contributing to society reflects empowerment and identity. Moreover, connectedness, a key CHIME component, strongly shaped decisions. Individuals who feared losing social relationships expressed a reluctance to assume the risk of discontinuation, underscoring the significance of social ties in shaping their illness perceptions.

Most participants reported a good relationship with their clinical team and satisfaction with their involvement in antipsychotic decisions. However, few had had explicit discussions about discontinuing medication and, when it was mentioned, it was usually initiated by the participants themselves. This aligns with previous studies where individuals with psychotic disorders also reported limited involvement in such discussions.^29,33^ Several factors could explain this: participants may not recall the conversations, these may not have occurred or clinicians may have been unclear or assumed that patients preferred to continue treatment, deeming the topic unnecessary.

A key factor influencing participants’ decisions was the perception of medication as a symbol of illness or abnormality, directly tied to self-stigma – a finding echoed in other studies.^34,35^ Notably, only one participant in our study used long-acting injectables (LAIs), and research suggests that these may not act as a constant reminder of illness, unlike oral medications.^36^ Given the potential impact of this factor on decision-making, it would be valuable to explore whether LAI use could shift participants’ perceptions and influence their decisions about continuing treatment.

### Limitations

Despite the valuable insights, our study has some limitations. Recruitment challenges resulted in a relatively homogeneous sample, particularly in terms of cultural diversity. This reflects both the characteristics of individuals referred by clinicians and the broader demographic composition of EIS. As a result, the transferability of our findings to more diverse populations may be limited. Although individuals with FEP are a hard-to-reach population, the study still offers critical insights into the complex decision-making process and the influence of personal motivators and external factors. Participants were clinician-referred, which may have led to a sample reflecting the perspectives of more compliant patients inclined to continue treatment. With only three participants discontinuing, their experiences may not have been fully explored, warranting further efforts. Nevertheless, our study covers multiple decision paths, because two participants were ambivalent and one had considered stopping before ultimately changing his mind. Additionally, specific psychosis diagnoses and co-medications were not collected, which may have influenced participants’ perspectives on stopping antipsychotics. Additionally, the use of online interviews – while necessary due to logistical constraints – may have influenced participants’ openness, and the absence of in-person interaction could have led to missed visual cues, potentially affecting the depth or interpretation of some responses. However, despite this limitation, participants in this study still shared very personal and meaningful experiences.

### Clinical and policy implications

Clinicians must engage individuals in remission from FEP in meaningful discussions about their personal motivators, because these shape how treatment information is processed. Clinicians should take a proactive and explicit approach, initiating clear conversations about treatment options, including the possibility of discontinuation, particularly during periods of illness stability. It is important for clinicians to provide information about the illness while also considering the individual’s own narrative explanation of their psychosis, rather than solely imposing a medical explanation.^37^ Balancing the risks of relapse with personal recovery goals, clinicians should provide information on both discontinuation and continuation of antipsychotics, discussing the potential consequences and strategies for managing relapse without inducing fear. Tailoring these conversations to each patient’s unique motivators, such as well-being or societal contribution, aligns treatment with patient values, strengthens the therapeutic alliance, empowers patients and may prevent unplanned treatment discontinuation.^38–40^

From a policy perspective, it is crucial to integrate these insights into clinical guidelines by ensuring that healthcare providers routinely discuss long-term medication management, including the option of antipsychotic discontinuation, with their patients. While current guidelines mention SDM,^4,5^ they offer limited detail on how to effectively implement this process, particularly for decisions about discontinuation. However, recent research offers promising strategies for advancing SDM in this context. A recent pilot study evaluating the Antipsychotic Medication Decision Aid (APM-DA) in a first-episode psychosis programme demonstrated the tool’s feasibility and usability in routine care.^41^ While further RCTs on antipsychotic discontinuation are needed to inform such tools, policies should also clarify the limited evidence on long-term outcomes and the associated uncertainties.^10^ Regularly reviewing the evolving motivators and influencing factors of patients ensures that the decision-making process remains personalised and aligned with their recovery goals. This comprehensive approach will enhance SDM quality, and may improve treatment outcomes and support patient-centred care.

In conclusion, fostering proactive, personalised care and updating clinical guidelines to strengthen SDM can better support individuals facing complex decisions about antipsychotic treatment. While existing decision aids provide some support,^28,42^ more tailored tools are needed for the FEP population to fully address the nuances of this decision.^20^ Along with stronger evidence, these advancements may improve patient outcomes and satisfaction in long-term mental healthcare.

## Supporting information

Béchard et al. supplementary material Béchard et al. supplementary material

## Data Availability

The data that support the findings of this study are available from the corresponding author, L.B., upon reasonable request.

## Supplementary material

The supplementary material is available online at https://doi.org/10.1192/bjo.2025.10817
