# Supplementary material for: Factors shaping the decision-making process to continue or discontinue antipsychotics: exploratory qualitative study of 12 individuals in remission from first-episode psychosis
Source: BJPsych Open. 2025 Sep 4;11(5):e193. doi: 10.1192/bjo.2025.10817 (PMC12451548; doi:10.1192/bjo.2025.10817)
Supplement: Béchard et al. supplementary material [file S205647242510817Xsup001.docx]

**Supplementary material 1: The Interview Guide**

This interview guide was used to explore participants’ decision-making processes regarding antipsychotic treatment. The guide is structured around key topics, with sample questions provided to facilitate discussion. The following outline provides a general idea of the topics covered during the interviews.

**Demographic Questions:**

1. **Age:** _________
2. **Gender:**
   □ Male (cisgender)
   □ Female (cisgender)
   □ Transgender (female-identified)
   □ Transgender (male-identified)
   □ Gender-fluid/Non-binary
   □ Prefer not to answer
3. **Marital Status:**
   □ Married
   □ Common-law
   □ Divorced
   □ Separated
   □ Widowed
   □ Single (never married)
4. **Highest level of education completed:**
   □ Elementary
   □ High School
   □ CEGEP (DEC)

□ Vocational degree
□ University (undergrad, postgrad, PhD, etc.)

1. **Primary Language**
   □ French
   □ English
   □ Other: ____________
2. **Race and National Origin:**
   □ White participant
   □ Black participant
   □ Asian participant
   □ Hispanic participant

□ Latino participant
□ Other: ___________

1. **Current occupation (check all that apply):**
   □ Employed
   □ Full-time student
   □ Part-time student
   □ Unemployed
   □ Other: ______________
2. **Current residence:**
   □ Independent living
   □ Living with parents
   □ Group home
   □ Other: ____________
3. **How long have you been under care in early intervention services?**
   ______ years _______ months
4. **Have you been hospitalized for psychiatric reasons?**
   □ Yes □ No
5. **If yes, when was your last hospitalization?**
   **Date : ___/___/___**
6. **How many times have you been hospitalized for psychiatric reasons?**
   ______ times
7. **Are you currently taking antipsychotic medication?**
   □ Yes □ No
8. **If yes, which one(s)?**
9. **How satisfied are you with your medication(s) on a scale of 1 to 10?**
   ______ (1 = very dissatisfied, 10 = very satisfied)
10. **Do you believe you have a mental health condition requiring antipsychotics?**
    □ Yes □ No □ Not sure □ Prefer not to answer

**Discussion on Medication:**

1. Can you describe how taking medication was first presented to you?
2. What are your personal thoughts on taking medication?
3. Describe your discussion with the team about stopping or continuing medication (when, context, options, how it was approached).
4. What are your family's and team's opinions on stopping or continuing medication?
5. What support did you receive for the decision?

Medication Continuation and Stopping:

1. Why do you take medication?
2. What are the pros and cons of continuing medication?
3. What are the risks and benefits of stopping medication?

Decision-Making Process:

1. What is most important and least important to you in this decision?
2. How would you like your team and family to be involved in the decision-making process?
3. What barriers or difficulties have you encountered when discussing stopping or continuing medication with your team or family?
